# A Systematic Review of β-Lactamase and Carbapenemase-Producing *Escherichia coli* at the Animal–Food Interface in the Middle East and North Africa

**DOI:** 10.3390/vetsci13090994

**Published:** 2026-09-20

**Authors:** Hamid Reza Sodagari, Ihab Habib

**Affiliations:** 1 Pathobiology Department, School of Veterinary Medicine, St. George’s University, St. George’s P.O. Box 7, Grenada; 2 Veterinary Public Health Research Laboratory, Department of Veterinary Medicine, College of Agriculture and Veterinary Medicine, United Arab Emirates University, Al Ain P.O. Box 15551, United Arab Emirates; 3 ASPIRE Research Institute for Food Security in the Drylands (ARIFSID), United Arab Emirates University, Al Ain P.O. Box 15551, United Arab Emirates

**Keywords:** antimicrobial resistance, *Escherichia coli*, extended-spectrum β-lactamase, carbapenemase, foodborne antimicrobial resistance, One Health

## Abstract

Antimicrobial resistance is a major global health challenge, and food-producing animals and foods of animal origin may contribute to the dissemination of resistant bacteria through the food chain. This review aimed to summarize recent evidence on extended-spectrum β-lactamase- and carbapenemase-producing *Escherichia coli* in food animals and animal-derived foods across Middle East and North Africa countries. A structured literature search was conducted to identify peer-reviewed studies published between 2016 and 2025. Data regarding country, sample type, prevalence, and resistance genes were extracted and analyzed descriptively. Twenty-eight studies from nine MENA countries revealed the presence of resistant *E. coli* in poultry, cattle, sheep, swine, camels, seafood, and other animal-derived products. Poultry showed a higher reported prevalence of extended-spectrum β-lactamase-producing *E. coli*, while carbapenem-resistant isolates were less frequently reported but were detected in several countries. The *bla*_CTX-M_ family was the predominant extended-spectrum β-lactamase determinant, whereas *bla*_NDM_, *bla*_OXA-48_-like, and *bla*_KPC_ were among the commonly identified carbapenemase genes. These findings demonstrate that food animals and animal-derived foods may represent important reservoirs of clinically relevant resistance genes in the Middle East and North Africa region. Strengthening One Health surveillance, antimicrobial stewardship, and regional collaboration is essential to limit the spread of resistant bacteria.

## Simple Summary

Antimicrobial resistance is a growing global health problem that makes infections in humans and animals more difficult to treat. Food-producing animals and foods derived from animals may serve as potential reservoirs or vehicles for bacteria carrying antimicrobial resistance traits, which could contribute to the dissemination of resistance through the food production chain. This review examined published studies from the Middle East and North Africa region over the last 10 years to assess the occurrence of antimicrobial-resistant *Escherichia coli* in food animals and animal-derived foods. The findings showed that resistant bacteria have been reported in several countries, with poultry and seafood products identified as important sources of resistant *E. coli*. Genes associated with resistance to commonly used antibiotics were frequently detected, including genes that can make bacteria resistant to some of the most important medicines used for treating serious infections. These findings highlight the need for improved monitoring of antimicrobial resistance in food production systems and stronger collaboration between human, animal, and environmental health sectors. Enhanced surveillance and responsible antimicrobial use in animal production are essential to limit the emergence and spread of resistant bacteria and protect public health.

## 1. Introduction

Antimicrobial resistance (AMR) is a serious and growing global challenge influencing human and veterinary medicine [1,2]. *Escherichia coli* (*E. coli*) is a ubiquitous organism found in animals, humans, and the environment [3]. It is widely used as an indicator for monitoring AMR in the food chain due to its remarkable ability to acquire and disseminate diverse resistance determinants [4]. The emergence of extended-spectrum β-lactamase (ESBL) and carbapenem-producing *E. coli* is a serious public health concern [5]. Such multidrug-resistant bacteria can be transmitted to humans from animal sources through the food chain [5]. Numerous previous studies have reported high rates of *E. coli* producing ESBLs or carbapenemases in food animals and animal-derived food [6,7,8,9].

Antimicrobial resistance is driven by several factors, including high levels of antibiotic usage in both human and veterinary medicine, insufficient regulation of antimicrobial use in some parts of the world, and gaps in continuous monitoring and surveillance systems [10]. In addition, several Middle East and North Africa (MENA) countries are highly dependent on imported food products, including meat, poultry, and dairy, due to population growth and negative impacts of climate change on agricultural capacity [11]. This reliance on international food supply chains may facilitate the introduction and dissemination of antimicrobial-resistant bacteria, including ESBL- and carbapenemase-producing *E. coli*, originating from regions with varying antimicrobial stewardship and surveillance standards [12]. Consequently, imported foods represent an additional and often under-recognized pathway for the transboundary spread of AMR in the MENA region [12].

Food-producing animals and their products in the MENA region can be an overlooked yet significant pathway for resistant bacteria, including ESBL and carbapenem-producing *E. coli*. These food animals have the potential to transfer resistant pathogens to humans either directly or indirectly through the food chain [13]. Despite such risk, data from the MENA region remain fragmented and country-specific. This review aims to collect recent findings (over the last 10 years) on ESBL- and carbapenemase-producing *E. coli* in food animals and animal-derived food across MENA countries, offering an updated picture of its prevalence, genetic determinants, and distribution across sample types. Unlike previous regional reviews that have examined antimicrobial resistance in foodborne pathogens more broadly or have focused on other pathogen-specific or geographic scopes [14,15,16], this review specifically focuses on ESBL- and carbapenemase-producing *E. coli* in food animals and foods of animal origin across MENA countries. By addressing a region that is highly interconnected through international food trade [17] and human mobility [18], this synthesis provides insights of global relevance for understanding the transboundary dissemination of antimicrobial resistance and for informing One Health–based surveillance and control strategies.

## 2. Materials and Methods

### 2.1. Review Design and Eligibility

A systematic review was conducted to characterize β-Lactamase -producing and carbapenem-resistant *Escherichia coli* recovered from food-producing animals and animal-derived foods in the MENA region. This review was performed in accordance with the PRISMA (Preferred Reporting Items for Systematic Reviews and Meta-Analyses) guidelines [19,20]. Reporting followed PRISMA 2020 and PRISMA-S [19,20], and the completed PRISMA 2020 checklist is provided in the Supplementary Materials (File S1). Eligible reports were peer-reviewed original studies published in English from 1 January 2016 to 31 December 2025 that sampled poultry, ruminants, camels, swine, or aquatic food species, or meat, milk, eggs, fish, shellfish, and related food matrices; identified *E. coli* by culture-based, biochemical, mass-spectrometric, or molecular methods; and reported an ESBL phenotype, carbapenem non-susceptibility, or an ESBL/carbapenemase gene. The operational region comprised Algeria, Bahrain, Egypt, Iraq, Israel, Jordan, Kuwait, Lebanon, Libya, Morocco, Oman, Palestine, Qatar, Saudi Arabia, Syria, Tunisia, the United Arab Emirates, and Yemen.

Reviews, editorials, conference abstracts without a full article, experimental challenge studies lacking field or food-chain sampling, reports limited to humans or non-food animals, studies outside the region or date range, and reports without an eligible *E. coli* resistance outcome were excluded. For overlapping populations, the most complete report was retained. Studies without an exact prevalence denominator remained eligible for qualitative evidence mapping but were not pooled.

### 2.2. Information Sources, Search, and Study Selection

**Figure 1:**
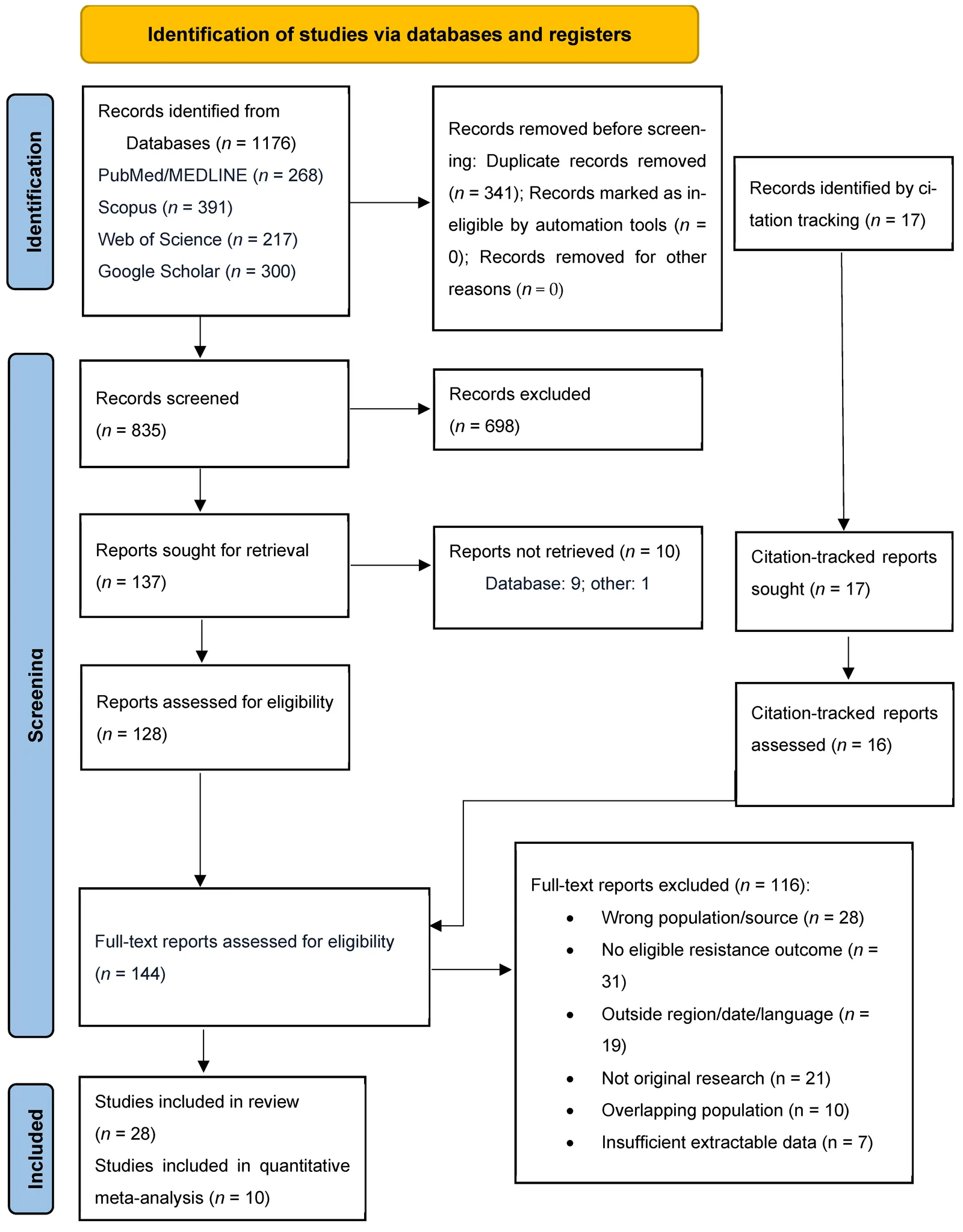
PRISMA procedural flow diagram of the literature search and study selection process.

PubMed/MEDLINE, Scopus, Web of Science Core Collection, and Google Scholar were searched for records published during 2016–2025. The search combined organism terms (“*Escherichia coli*” OR “*E. coli*”), resistance terms (ESBL OR “extended-spectrum beta-lactamase” OR carbapenem* OR carbapenemase OR *bla*_CTX-M_ OR *bla*_TEM_ OR *bla*_SHV_ OR *bla*_OXA_ OR *bla*_NDM_ OR *bla*_KPC_ OR *bla*_VIM_ OR *bla*_IMP_), animal/food-source terms, and each country name. No study-design filter was applied. The first 300 Google Scholar results sorted by relevance were assessed as a supplementary search, and backward and forward citation tracking was performed for included reports. Search reporting and record accounting followed PRISMA-S [20]. Database records were exported and deduplicated using DOI, PMID, title, first author, and year, followed by manual review of near-duplicate titles. Two reviewers independently screened titles and abstracts, retrieved potentially eligible full texts, and applied the same prespecified criteria at full-text review; disagreements were resolved by consensus. One mutually exclusive primary reason was assigned to each full-text exclusion. The reconstructed selection flow comprised 1176 database records and 17 citation-tracked records, 341 duplicates, 835 title/abstract records screened, 144 full texts assessed, 116 full-text exclusions, and 28 included studies (Figure 1).

### 2.3. Data Extraction, Harmonization, and Quality Appraisal

A standardized form captured country, year, host or food source, sample matrix, sample size, number of *E. coli* isolates, ESBL-positive isolates, phenotypic carbapenem resistance, ESBL genes, carbapenemase genes, and reference identifier. Extraction was cross-checked against the full report. Source descriptions were harmonized into poultry gut/hatchery, poultry meat/carcass, poultry clinical/tissue, ruminant gut/fecal, ruminant food/clinical, seafood product/tissue, swine fecal, and mixed animal/food strata. Reported β-lactamase genes were grouped at the family level (CTX-M, TEM, SHV, and OXA) when reported in ESBL-focused studies; these gene families were not considered evidence of an ESBL phenotype unless the study reported a specific ESBL-associated allele and/or phenotypic confirmation. Carbapenemase genes were grouped as NDM, KPC, VIM, IMP, and OXA-48-like; *bla*OXA-48 and *bla*OXA-181 were combined as OXA-48-like carbapenemases. A gene not reported in a study was not treated as a confirmed negative.

Each report contributing a prevalence estimate was independently appraised by two reviewers using the nine-item Joanna Briggs Institute Critical Appraisal Checklist for Studies Reporting Prevalence Data [21]. The checklist evaluates the appropriateness of the sampling frame and sampling method, sample-size adequacy, description of the study population and setting, coverage of the analyzed sample, validity and consistency of outcome measurement, statistical analysis, and response or participation. Each item was classified as yes, no, unclear, or not applicable, and disagreements were resolved by consensus. Because the checklist does not prescribe a validated numerical threshold, no composite quality score or post hoc low-, moderate-, or high-quality classification was applied. Studies were not excluded solely on the basis of appraisal; instead, domain-level findings were used to qualify the synthesis and interpretation [21]. Reports retained solely for qualitative evidence mapping were characterized descriptively when prevalence-specific appraisal items were not applicable.

### 2.4. Evidence Mapping and Meta-Analysis

Country–source/sample–gene networks used the study report as the counting unit. Link width represents the number of reports contributing a detection link; because a report could contribute to several gene families, the networks do not estimate prevalence or establish within-isolate co-occurrence. Quantitative synthesis was restricted to reports with an exact ESBL-positive *E. coli* numerator and an exact *E. coli*-isolate denominator. Sample-level denominators, irrecoverable counts, and internally inconsistent count–percentage pairs were excluded. Ten records from six countries met these criteria. Formal subgroup analysis or meta-regression by country, animal host, food matrix, or sampling method was not performed because only ten studies were available, several country–source combinations were represented by a single study, and country was strongly confounded with host or matrix, health status, isolate-selection procedure, and laboratory method. Under these conditions, regression coefficients would be unstable and could not reliably separate methodological effects from genuine epidemiological variation.

Study prevalence was calculated as the number of ESBL-positive *E. coli* isolates divided by the number of *E. coli* isolates tested, with study-specific 95% Wilson confidence intervals. The analysis was performed using Python 3.12.13 (Python Software Foundation, Wilmington, DE, USA), with NumPy 2.3.5 (NumPy Developers, USA), SciPy 1.17.0 (SciPy Developers, USA), and Matplotlib 3.10.8 (Matplotlib Development Team, USA). For study *I*, with *xi* ESBL-positive isolates among *ni E. coli* isolates, the observed proportion was transformed as *yi = log[xi/(ni − xi)]*, with within-study variance *vi = *1*/xi + *1*/(ni − xi)*. A continuity correction of 0.5 was prespecified for both cells when *xi* equaled 0 or *ni*; none of the studies included in the final analysis required this correction. Between-study variance, τ^2^, was estimated by minimizing the negative restricted log-likelihood using the bounded scalar optimizer implemented in scipy.optimize.minimize_scalar [22]. Optimization was performed over τ^2^ values from 0 to 100 on the logit-variance scale, with a convergence tolerance of 1 × 10^−12^. Random-effects weights were calculated as *wi = *1*/(vi + τ^2^)*, and the pooled logit was obtained as $\hat{\mu }$ *= Σwiyi/Σwi*.

For the modified Hartung–Knapp–Sidik–Jonkman procedure, q was calculated as *Σwi(yi − μ)^2^/(k − *1*)*, and the variance of the pooled logit was defined as *max(*1*, q)/Σwi* [23]. The pooled 95% confidence interval used the t distribution with *k − *1 degrees of freedom. The 95% prediction interval was calculated on the logit scale as $\hat{\mu }$ ± t0.975, k − 2√(τ^2^ + SEHKSJ^2^). Pooled estimates, confidence limits, and prediction limits were subsequently back-transformed using the inverse-logit function [23]. Cochran’s Q was calculated using fixed-effect inverse-variance weights, and I^2^ was calculated as *max [*0*, (Q − k + *1*)/Q] × *100%. In the leave-one-out analysis, each study was omitted sequentially and τ^2^ and the pooled estimate were re-estimated from the remaining studies [24].

Quantitative pooling was not performed for carbapenem-resistant *E. coli* because there were insufficient numbers of sufficiently comparable studies to support a meaningful pooled estimate. Carbapenem-resistance and carbapenemase findings were therefore synthesized descriptively.

## 3. Results

Our findings showed that, among MENA countries, eligible studies reporting ESBL- and/or carbapenem-resistant *E. coli* in food animals and animal-derived foods were identified from nine countries: Tunisia, Egypt, Palestine, Lebanon, Algeria, UAE, Qatar, Saudi Arabia, and Morocco. Considerable variation in sample size was observed, from a small-scale study on oysters (33 isolates) [8] to large cross-sectional studies on seafood (1716 isolates) [25]. The examined sample types included fecal samples from cattle, camel, sheep, poultry, turkey, and swine, as well as animal-derived food products. Table 1 shows the summary of the relevant studies published on ESBL- and carbapenemase-producing *E. coli* in food animals and animal-derived food across MENA countries over the last 10 years.

### 3.1. Methodological Quality Appraisal

The appraisal indicated greater confidence in laboratory outcome ascertainment than in the representativeness and precision of the prevalence estimates. Study sources and settings were generally described, and *E. coli* identification and ESBL or carbapenemase characterization usually employed recognized phenotypic and/or molecular methods. The principal recurring concerns involved non-probability or geographically restricted sampling, absence of priori sample-size justification, and isolate-level analyses that did not account for clustering by the epidemiological sampling strata (e.g., farm, flock, or retail site). Confidence intervals and information concerning farm or site participation and nonresponse were also rarely reported.

Among the ten studies retained in the quantitative synthesis after denominator verification, four used cephalosporin-containing selective media before ESBL confirmation [6,7,9,26]. Their reported proportions therefore represent ESBL positivity among resistant-screened isolates rather than prevalence among all *E. coli* in the sampled population. Two studies were restricted to clinically affected animals [27,28], and five included fewer than 100 *E. coli* isolates [8,9,26,28,29]. The remaining quantitatively synthesized studies used nonselective isolation but were confined to particular farms or retail settings [8,29,30]. Accordingly, the appraisal identified important limitations in external validity, comparability, and statistical precision, despite generally appropriate microbiological identification and resistance-confirmation procedures. No study was excluded solely because of its quality appraisal.

### 3.2. Prevalence of ESBL-Producing E. coli Across MENA Region

ESBL-producing *E. coli* were identified in a wide range of food animal and foods of animal origin samples. The lowest reported prevalence was detected in seafood products, including farmed fish (9/641, 1.4%) and clams (14/1075, 1.6%) in a Tunisian study [25], whereas the highest reported estimate (114/120, 95%) was reported in broiler chicken cecal contents from the UAE [6]. Our review also found that 80 of 111 *E. coli* isolates recovered from poultry cecal samples in Tunisia (72.1%) were ESBL-positive, with the isolates exhibiting high resistance rates to multiple β-lactam antibiotics [7]. A study from the UAE reported that nearly 80% of retail chicken carcasses were contaminated with ESBL-producing *E. coli* [31]. In Egypt, 65% of *E. coli* isolates recovered from turkeys (17/26) and broiler chickens (92/140), and 45% of isolates obtained from oysters (11/24), were identified as ESBL producers [8,26,27]. Furthermore, 36.6% (26/71) of *E. coli* isolates from diarrheic calves in Tunisia were ESBL-positive in a 2023 study [28]. Studies from Lebanon also reported considerable prevalence, with ESBL-producing *E. coli* detected in 28% of poultry fecal isolates and 88% of swine fecal isolates [32,33]. An Algerian study likewise reported a high prevalence of multidrug-resistant *E. coli* isolates from cattle and sheep feces, with resistance to multiple β-lactam antibiotics [9].

Overall, the highest reported study-level estimates were frequently observed in poultry, although substantial variation was evident across animal and food sources. These findings indicate that ESBL-producing *E. coli* have been reported across multiple food-animal and food-source categories in the MENA region.

### 3.3. Prevalence of Carbapenem-Resistant E. coli Across the MENA Region

Carbapenem-resistant *E. coli* were reported less frequently than ESBL-producing isolates across the MENA region. In Algeria, 52% of *E. coli* isolates recovered from sheep and cattle were resistant to ertapenem, while 41% exhibited resistance to imipenem [9]. Similarly, high prevalence rates (50%) of carbapenem-resistant *E. coli* were reported in Egypt from broiler chickens [27], oysters [8], and cattle affected by secondary infections after a Foot and Mouth Disease (FMD) outbreak [34]. Other Egyptian studies reported variable carbapenem resistance rates, ranging from 0% resistance in turkeys [26] to 50% resistance to ertapenem among *E. coli* isolates recovered from broiler lungs [27]. Tunisian studies reported a moderate prevalence of carbapenem-resistant *E. coli* (20%) [7], whereas other investigations reported no or very low levels of carbapenem resistance.

### 3.4. ESBL and Carbapenemase Resistance Genes

**Figure 2:**
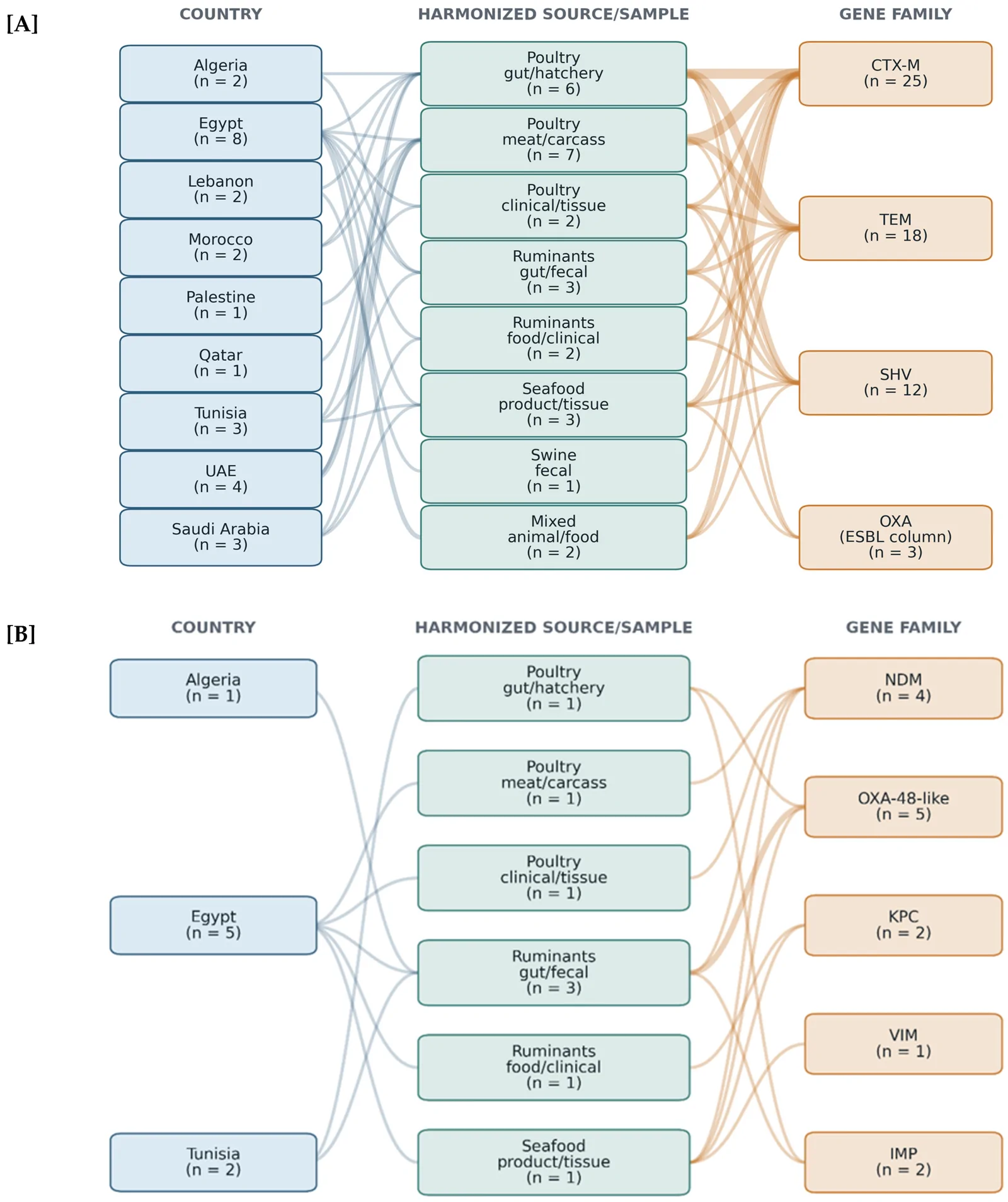
Country–source/sample– in relation to (**A**) β-lactamase and (**B**) carbapenemase gene-family relationships in the 28-study evidence base. Link width represents the number of study reports contributing to each connection; a report may contribute to more than one gene-family link.

Among screened study records reporting at least one β-lactamase gene family, CTX-M was the dominant family (25 records) and connected all nine represented countries with all eight harmonized source/sample strata. TEM and SHV were reported in 18 and 12 records, respectively. The most frequent study-level connections involved poultry gut/hatchery and poultry meat/carcass sources (Figure 2A).

Carbapenemase genes were reported in eight records from Algeria, Egypt, and Tunisia. OXA-48-like enzymes were most frequent (five records), followed by NDM (four), KPC and IMP (two each), and VIM (one). The links spanned poultry, ruminant, and seafood matrices; Egypt contributed the greatest source diversity and four of the five represented carbapenemase families, whereas IMP was confined to Tunisian records (Figure 2B).

### 3.5. Evidence Mapping and Meta-Analysis of ESBL-Positive E. coli

**Figure 3:**
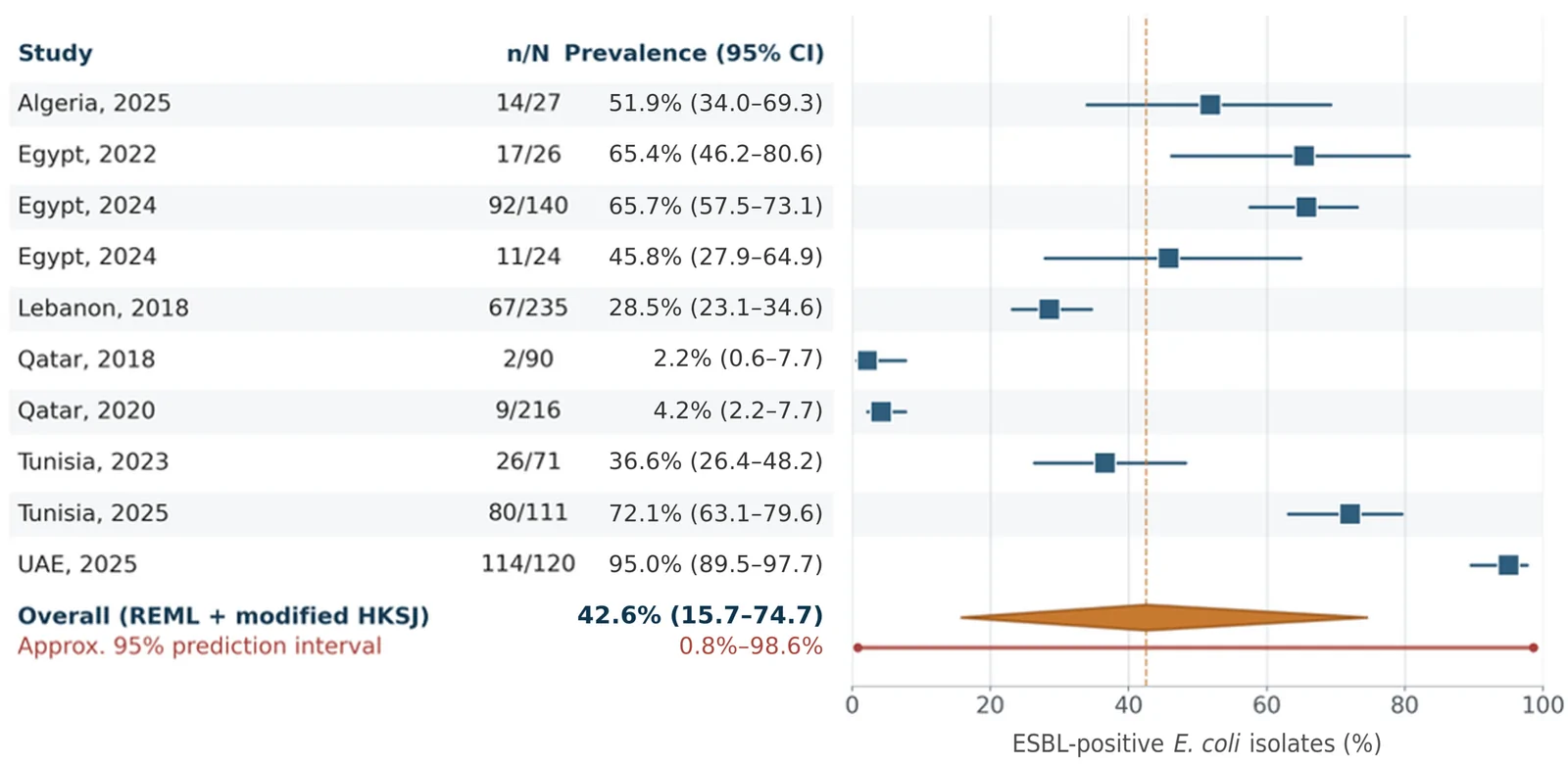
Exploratory random-effects meta-analysis of ESBL-positive *E. coli* isolates across countries and food-related sources in the MENA region. Study data, in figure order, were obtained from Kirat et al. [9], Moawad et al. [26], Ali et al. [27], Mohammed et al. [8], Dandachi et al. [33], Eltai et al. [29,30], Ben Haj Yahia et al. [28], Tayh et al. [7], and Khalifa et al. [6]. Squares show study estimates scaled by random-effects weight, horizontal lines show Wilson 95% confidence intervals, the diamond shows the pooled estimate, and the red line shows the prediction interval.

Ten records from six countries were quantitatively synthesized for an illustrative pooled ESBL-positive *E. coli* (Figure 3). The distribution of study characteristics was sparse and unbalanced across countries. Countries did not contribute comparable sets of animal hosts or food matrices, and sampling and isolate-selection methods also varied within and between countries. Country, source, and methodological effects could therefore not be evaluated independently. Quantitative synthesis was limited to screened studies with an exact ESBL-positive *E. coli* numerator and an exact *E. coli*-isolate denominator. Sample-level denominators, irrecoverable counts, and internally inconsistent count–percentage pairs were excluded. Study prevalence estimates ranged from 2.2% to 95.0%, and the random-effects pooled estimate was 42.6% (95% CI 15.7–74.7%). Heterogeneity was substantial (Q = 239.3, df = 9, I^2^ = 96.2%, τ^2^ = 3.48), with a 95% prediction interval of 0.8–98.6%. Leave-one-out pooled estimates ranged from 34.4% to 51.1%.

**Table 1:** ESBL- and carbapenemase-producing *E. coli* in food animals and animal-derived food across MENA countries (2016–2025).

Country	Year	Source	Sample Type	Sample Size	*E. coli* (n. Isolates (%))	β-Lactam Resistance (%)	ESBL-Producing *E. coli* (n. Isolates (%))	ESBL Genes Detected	Carbapenem Resistance (%)	Carbapenemase Genes Detected	References
Algeria	2025	Cattle and sheep	Fecal samples	285 (cattle n = 145; sheep n = 140)	- ^1^	Cefotaxime (100) Cefepime (100) Aztreonam (100) Amoxicillin-clavulanic acid (100) Amoxicillin (100) Ceftazidime (85) Cefoxitin (48)	14/27 (52%)	*bla*_CTX-M_	Ertapenem (52) Imipenem (41)	*bla*_NDM-1_ *bla*_OXA-181_	[9]
Algeria	2025	Day-old broiler and layer chicks	Pooled samples from hatcheries	100	68/100 (68%)	-	Broiler 11/100 (11%) Layer 2/100 (2%)	*bla*_CTX-M-1_ *bla*_CTX-M-14_	-	-	[35]
Egypt	2016	Dairy cattle	Rectal swabs	266	-	-	114/266 (42.8%)	*bla*_CTX-M1/15_ *bla*_CTX-M9_ *bla*_TEM_ *bla*_SHV_	Imipenem Meropenem	*bla*_OXA-48_ *bla*_OXA-181_	[36]
Egypt	2017	Chicken and beef	Raw chicken and beef meat samples/internal organs	180	21/180 (11.7%)	Cefotaxime (33.3) Ampicillin (71.4) Amoxicillin-clavulanic acid (61.9)	-	*bla*_TEM-104_ *bla*_CTX-M-1_ *bla*_CTX-M-14_	-	-	[37]
Egypt	2020	Chicken	Retail chicken carcasses (neck skin)	145	-	Ampicillin Amoxicillin-clavulanic acid Cefotaxime Ceftazidime ceftriaxone,	-	*bla*_CTX-M-3_	Meropenem Imipenem	*bla*_NDM-1_ *bla*_NDM-5_	[38]
Egypt	2020	Cattle	Clinical samples from secondary infections following FMD outbreak	160	48/160 (30%)	Ampicillin (100) Amoxicillin (100) Amoxicillin-clavulanic acid (60.4) Cefotaxime (83.3) Ceftazidime (83.3)	-	*bla*_TEM_ *bla*_CTX_	Imipenem (50%) Meropenem (50%)	*bla*_KPC_	[34]
Egypt	2022	Turkeys	Cloacal swabs	250	26/250 (10.4)	Penicillin (100) Ceftazidime (62.5)	17/26 (65.4%)	*bla*_TEM_ *bla*_CTX-M9_ *bla*_SHV_ *bla*_OXA-10_	Imipenem (0%)	-	[26]
Egypt	2022	Cattle and poultry	Cattle rectal swabs, milk, poultry cloacal swabs	93	-	Cefotaxime Ceftazidime Cefepime Amoxicillin-clavulanic acid	30 isolates	*bla*_SHV_ *bla*_TEM_ *bla*_CTX-M_	Imipenem	-	[39]
Egypt	2024	Broiler chickens	Lung samples	250	140/250 (56%)	Cefotaxime (92.9) Ceftazidime (92.9) Ampicillin (96)	92/140 (65.7%)	*bla*_TEM-3_ *bla*_CTX-M-1_ *bla*_CTX-M-14_ *bla*_SHV-4_	Ertapenem (50%)	*bla*_NDM-1_	[27]
Egypt	2024	Oyster	Pooled fresh oyster samples	33	24/33 (72.7%)	Cefoxitin (83.3%)	11/24 (45.8%)	*bla*_TEM_ *bla*_CTX-M_ *bla*_SHV_ *bla*_OXA-1_	Meropenem (50%) Ertapenem (50%)	*bla*_KPC_ *bla*_NDM_ *bla*_OXA-48_ *bla*_VIM_	[8]
Lebanon	2018	Poultry	Fecal swabs	981	217 isolates of the 235 strains isolated (92%)	Ampicillin (100) Cefotaxime (90) Ceftazidime (55) Cefoxitin (48) Cefepime (14) Amoxicillin-clavulanic acid (35)	67 (28.5%)	*bla*_TEM_ *bla*_CTX-M_ *bla*_SHV_	-	-	[33]
Lebanon	2019	Swine	Fecal samples	114	94.5%	-	98 isolates	*bla*_CTX-M_	-	-	[32]
Morocco	2025	Poultry	Poultry products	154	96/154 (62.34%)	-	-	*bla*_CTX-M-65_ *bla*_TEM-1_	-	-	[40]
Morocco	2025	Broiler chickens	Intestinal samples	216	83.5%	Ceftazidime (37) Amoxicillin (94) Ceftazidime (81)	10.32%	*bla*_CTX-M-15_ *bla*_TEM-1_	-	-	[41]
Palestine	2025	Broiler chickens	Clinical samples from necropsied chickens	65	65 avian pathogenic *E. coli* isolates	-	-	*bla*_TEM_ *bla*_CTX-M_ *bla*_OXA_ *bla*_SHV_	-	-	[42]
Qatar	2018	Broiler chickens	Fecal samples	172	90/172 (52.3%)	Ampicillin (72.2) Cefuroxime (4.4) Cephalothin (15.5) Ceftriaxone (2.2) Cefepime (1.1)	2/90 (2.2%)	-	-	-	[29]
Qatar	2020	Chicken	Retail chicken carcasses	270	216/270 (80%)	Ampicillin (52.3) Cephalothin (45.4) Amoxicillin-clavulanic acid (6) Ceftriaxone (5.1) Cefepime (2.3)	9/216 (4.2%)	*bla*_CTX-M-G1_ *bla*_TEM_ *bla*_SHV_ *bla*_CTX-M-G2_	Meropenem (1.4%) Ertapenem (0.9%)	-	[30]
Tunisia	2022	Seafood	Farmed fish, Mediterranean clams	1716	-	-	Fish: 9/641 (1.4%) Clams: 14/1075 (1.3%)	*bla*_CTX-M-15_ *bla*_CTX-M-1_ *bla*_CTX-M-14_ *bla*_CTX-M-27_	-	-	[25]
Tunisia	2023	Diarrheic calves	Fecal samples	100	71 isolates	-	26/71 (36.6%)	*bla*_CTX-M_ *bla*_SHV_ *bla*_TEM_	-	*bla*_OXA-48_ *bla*_IMP_	[28]
Tunisia	2025	Broiler chickens	Cecal samples	111	111 isolates	Amoxicillin (100) Cephalothin (96.4) Cefotaxime (83.8) Cefepime (82) Ceftazidime (75.7) Cefuroxime (73.9)	80/111 (72.1%)	*bla*_CTX-M-G1_ *bla*_TEM_ *bla*_SHV_	Ertapenem (20)	*bla*_OXA-48_ *bla*_IMP_	[7]
UAE	2025	Broiler chickens	Cecal samples	77	120 (82.9%) out of 146 Gram-negative isolates	Ampicillin (100) Ceftriaxone (91.1) Cefoperazone (89) Cefoxitin (48.6) Amoxicillin-clavulanic acid (10.3)	114/120 (95%)	*bla*_TEM_ *bla*_CTX-M-55_ *bla*_CTX-M-15_ *bla*_CTX-M-8_	Meropenem (0%), and Imipenem (0%)	-	[6]
UAE	2025	Broiler chicken	Frozen broiler chicken carcasses	253	248/253 (98%)	Ampicillin Cefotaxime Ceftriaxone Cefepime Cefoxitin	-	*bla*_CTX–M–55_ *bla*_CTX–M–8_	Imipenem (0%)	-	[12]
UAE	2024	Chicken	Chicken meat					*bla*_CTX-M-55_	-	-	[43]
UAE	2023	Chicken	Retail chicken carcasses	315	79.68%	Cefepime (46%)	79.68%	*bla*_TEM_ *bla* _CTX-M-55_ *bla*_CTX-M-15_	-	-	[31]
Saudi Arabia	2016	Camel	Cecal contents	100	26/100 (26%)	Ampicillin (50) Amoxicillin (23.1) Cefotaxime (7.7) Cefepime (3.8)	5/26 (19.2%)	-	Ertapenem (0) Imipenem (0) Meropenem (0)	-	[44]
Saudi Arabia	2018	Food	Beef meat samples	540	120/540 (22%)	Ampicillin (75) Amoxicillin-clavulanic acid (100) Penicillin (100)	-	*bla*_TEM_ *bla*_SHV_	-	-	[45]
Saudi Arabia	2016	Fish	Frozen Freshwater Fish	405	-	Cefotaxime Ceftazidime Ceftriaxone Cefepime Aztreonam	110/405 (27.2%)	*bla*_CTX-M_ *bla*_SHV_	-	-	[46]
Saudi Arabia	2021	Poultry	Poultry meat	40	-	Ampicillin Cephalothin Ceftazidime Ceftriaxone Cefepime	-	*bla*_CTX-M-15_ *bla*_TEM-1B_ *bla*_TEM-214_ *bla*_TEM-206_ *bla*_TEM-141_	-	-	[47]

^1^ Not reported.

## 4. Discussion

This review demonstrates that ESBL– and carbapenemase-producing *E. coli* are distributed across food animals and foods of animal origin across some of the MENA countries. The highest reported occurrence was observed in poultry compared to other food animals. This may suggest that poultry production systems are important reservoirs and potential sources for the dissemination of these bacteria in the MENA region [27,48]. In addition, seafood and aquatic environments can also be other sources for resistance genes [8]. ESBL and carbapenemase genes were reported in oysters from Egypt [8], whereas lower frequencies of these resistance genes were reported in fish and clams from Tunisia [25]. Ruminants and swine also carried ESBL and carbapenem-resistant *E. coli* isolates, which indicates their potential role in maintaining and disseminating AMR in the MENA region [9,32].

The dominance of *bla*_CTX-M_ genes in ESBL-producing *E. coli* derived from food animals across the MENA countries reflects global patterns. This gene family is recognized as the most important driver of ESBL resistance in both veterinary and human settings [49]. Historically, TEM- and SHV-type enzymes were the dominant ESBLs worldwide; however, this pattern has shifted, with CTX-M enzymes now being the most prevalent ESBL group [50]. CTX-M-producing *E. coli* have expanded from healthcare settings into community, animal, and environmental reservoirs, representing a major global AMR concern [51]. Among CTX-M variants, CTX-M-15 is the most widely distributed globally across human, animal, and environmental reservoirs [52,53]. The presence of other genes, including *bla*_TEM_, *bla*_SHV_, and *bla*_OXA_ variants, further broadens the ESBL-resistance spectrum. In food-producing animals, ESBL-associated genes, including *bla*_TEM_, *bla*_SHV_, and *bla*_CTX-M_, are commonly detected among *Enterobacteriaceae* isolates [54].

The broad CTX-M connectivity across countries and food-animal interfaces indicates that ESBL surveillance cannot be confined to a single commodity or production stage. Although the study-level links do not demonstrate transmission, their cross-sector distribution supports coordinated One Health surveillance that couples farm, slaughter, retail-food, and environmental sampling with allele-level and genomic characterization to distinguish shared reservoirs from independent selection events. Detection of clinically important carbapenemases at animal and food interfaces is relevant even when the number of reports is small because these genes can occur on mobile genetic platforms shared across human, animal, food, and environmental compartments [55]. Their concentration in three countries may reflect both true local emergence and unequal testing intensity, emphasizing the need for harmonized regional surveillance, confirmatory molecular testing, and linkage of veterinary findings with human and wastewater antimicrobial-resistance programs.

Although carbapenemase-producing *E. coli* were less common than ESBL producers, their presence poses significant public health concerns. Carbapenemase-producing microorganisms are a major public health threat because they can confer resistance to carbapenems, which are important last-line antimicrobial agents for treating multidrug-resistant Gram-negative infections [56]. Carbapenemase-producing bacteria have been detected in livestock in the MENA region and elsewhere [8,9,57]. Their emergence may result from antimicrobial selection pressure and transmission from human, animal, or environmental sources [57]. Consequently, animal-derived products may represent potential sources of carbapenemase-producing bacteria, highlighting a possible food-chain pathway for their dissemination to humans [58].

The detection of *bla*_NDM_ and *bla*_OXA_ variants (OXA-48, OXA-181) across food animals from Algeria, Egypt, and Tunisia underscores the possible dissemination of these genes to humans through the food chain. The co-occurrence of other carbapenemase genes, including *bla*_KPC_ and *bla*_VIM_ in oysters from Egypt, demonstrates the risk posed by the aquatic environment, representing seafood as one of the important reservoirs of carbapenemase genes [59]. Furthermore, the present review identified *bla*_IMP_ in poultry from Tunisia, consistent with reports from other countries worldwide [60,61]. The major clinically relevant carbapenemases belong to five key families: KPC, OXA-48-like, NDM, VIM, and IMP [57]. KPC and OXA-48-like enzymes are serine carbapenemases, whereas NDM, VIM, and IMP are metallo-β-lactamases [56,62]. Their distribution varies geographically; KPC, OXA-48-like, NDM, and VIM are widely reported in Europe [63], while IMP enzymes are more prevalent in parts of East Asia and Australia [64]. All of these carbapenemase families have been detected in food-producing animals and their products in studies from the MENA region included in this review, highlighting the potential role of animal reservoirs and food chains in the dissemination of carbapenem resistance.

The high heterogeneity (I^2^) estimates across the pooled studies, together with the very wide prediction interval, indicate that a single pooled value does not convincingly describe a uniform regional estimate. The observed heterogeneity may reflect differences in animal and food sources, sampling frames, isolate selection, and laboratory procedures, as well as genuine epidemiological differences in AMR prevalence between countries. Because these methodological and epidemiological sources of variation could not be disentangled in the available studies, the pooled estimate should be interpreted as an exploratory summary rather than a uniform regional prevalence estimate.

The methodological appraisal elaborated on some of the unusually high study-level estimates. Antimicrobial-supplemented culture media preferentially recover resistant organisms; consequently, proportions calculated among isolates recovered after selective culture are conditional estimates and are not directly comparable with estimates based on all *E. coli* recovered through nonselective culture. Estimates derived from clinically affected animals may likewise be higher than those observed in healthy production populations. Restricted geographic sampling, small isolate denominators, and failure to account for clustered sampling further limit precision and generalizability. These considerations, together with the substantial statistical heterogeneity, mean that high study-level percentages and the pooled estimate should not be interpreted as national or regional population prevalence.

Overall, this review article highlights the potential public health threat posed by ESBL- and carbapenemase-resistant *E. coli* derived from food animals and foods of animal origin in some MENA countries. This finding emphasizes the need for reinforced One Health surveillance systems. Strengthening regulations and antimicrobial stewardship in food-animal production in the region would be advisable to help address antimicrobial resistance and protect the effectiveness of antimicrobials critical to human medicine.

This study has several limitations. Most available data were derived from a limited number of MENA countries, with no eligible studies identified from several countries. Differences in sample size, sample matrices, microbiological methods, antimicrobial susceptibility testing approaches, gene detection methods, variations in susceptibility breakpoints, study populations, and sampling representativeness complicate direct comparison and may partially explain the variability in reported prevalence. Most included studies used convenience or purposive rather than population-based probability sampling, introducing selection bias that may have preferentially captured higher-risk populations, inflated prevalence estimates, and limited their generalizability. Furthermore, English-language publications were only included in the present study, and relevant studies in other languages may have been missed. Therefore, the findings should be interpreted as reported occurrences of ESBL- and carbapenem-resistant *E. coli* rather than estimates of regional prevalence.

## 5. Conclusions

The present study underscores the occurrence of ESBL- and carbapenemase-resistant *E. coli* in food animals and foods of animal origin across nine different MENA countries. Several food-producing animals, including poultry, fish, swine, cattle, sheep, and camel, emerged as the reservoir of ESBL- and carbapenemase-producing *E. coli* in the region. The *bla*_CTX-M_ family was the commonly reported ESBL gene, while *bla*_NDM_ variants (NDM-1, NDM-5), *bla*_OXA_ variants (OXA-48, OXA-181), and *bla*_KPC_ were the reported Carbapenemase genes. Further studies are required to reinforce and complement our findings to better understand the transmission dynamics, sources, and potential public health impact of ESBL- and carbapenemase-producing *E. coli* in the MENA region. Distinguishing methodological variation from epidemiological disparities will require harmonized multicounty surveillance using comparable host populations, sampling frames, culture procedures, resistance definitions, and analytical methods.

## Data Availability

No new data were created or analyzed in this study.

## Supplementary Materials

The following supporting information can be downloaded at: https://www.mdpi.com/article/10.3390/vetsci13090994/s1, File S1: Completed PRISMA 2020 checklist.

## Abbreviations

The following abbreviations are used in this manuscript:

ESBL	Extended-Spectrum β-Lactamase
AMR	Antimicrobial resistance
MENA	Middle East and North Africa
